# Nutrients and Cardiometabolic Health in Type 2 Diabetes

**DOI:** 10.3390/nu15112517

**Published:** 2023-05-29

**Authors:** Domenico Tricò

**Affiliations:** Department of Clinical and Experimental Medicine, University of Pisa, Via Roma 67, 56126 Pisa, Italy; domenico.trico@unipi.it; Tel.: +39-050-99-3532

Paralleling the obesity pandemic, the prevalence and socioeconomic burden of type 2 diabetes are growing worldwide, requiring immediate attention and novel cost-effective preventive and therapeutic strategies. Type 2 diabetes is a multifaceted chronic disorder characterized by a complex interplay of genetic and environmental factors leading to impaired insulin secretion and reduced insulin sensitivity, ultimately resulting in elevated blood glucose levels. Chronic hyperglycemia, in turn, has established its role as a robust predictor of major cardiovascular outcomes, which can severely impair patients’ expectancy and quality of life [1].

Amidst this backdrop, nutrition therapy emerges as a crucial component in both the prevention and management of type 2 diabetes and its associated micro- and macrovascular complications [2]. Meal size and composition have a profound impact on the intricate physiological processes governing glucose homeostasis, especially in the earliest phase of the disease. Furthermore, recent insights extend beyond considerations of energy balance and nutrient composition, acknowledging the influential role of nutrient timing of consumption within a meal (e.g., food sequence, nutrient preloads [3,4,5]), as well as the daily distribution of energy and macronutrient intake (e.g., time-restricted feeding [6]), in shaping glucose metabolism and cardiovascular health [2]. Understanding the complex relationship between nutrition, glucose alterations, and associated cardiometabolic risk factors is essential for devising effective dietary strategies aimed at delaying the onset or mitigating the deleterious consequences of type 2 diabetes.

The purpose of this Special Issue of *Nutrients,* entitled “Nutrients and Cardiometabolic Health in Type 2 Diabetes”, was to gather contributions exploring the impact of nutrients and dietary patterns on glucose regulation and overall cardiometabolic health in individuals with or at risk for type 2 diabetes. This Special Issue features four original articles focusing on the effects of different dietary patterns on multiple cardiometabolic risk factors [7,8] and on the glucometabolic benefits of applying older and newer technologies in the treatment of people at risk for diabetes [9,10]. It also includes two review articles: one regarding the beneficial effects of aligning carbohydrate and energy consumption with the circadian clock [11], and one underscoring the relevance of gut peptides in the regulation of postprandial hemodynamic responses [12].

In recent years, there has been a gradual transition in research focus from examining the role of individual foods or nutrients on cardiometabolic health to evaluating dietary patterns [13]. Dietary patterns reflect the intricate combinations of foods and nutrients that are often consumed together and provide insights into a person’s long-term eating habits. In their article, Cao and colleagues [7] identified two common dietary patterns in a large cohort of adults from India with an adverse risk profile for atherosclerotic cardiovascular disease: a “snack-fruit” pattern and a “rice-meat-refined wheat” pattern. None of these patterns could be clearly defined as “healthy” or “unhealthy”, as each of them differently contributed to increase specific cardiometabolic risk factors: the “snack-fruit” pattern was longitudinally associated with increased triglycerides, while the “rice-meat-refined wheat” pattern was associated with central obesity and elevated glycated hemoglobin. These results highlight the challenges often encountered in nutrition research, emphasizing the importance of moving away from a narrow examination of isolated nutrients and towards a broader understanding of individuals’ dietary habits. By adopting this wider perspective, healthcare professionals can better tailor dietary interventions based on the individual’s cardiometabolic risk profile.

A Mediterranean-style dietary pattern with balanced macronutrient composition is recommended for the management of obesity and associated cardiometabolic risk factors. Nevertheless, low-carbohydrate, high-protein diets have been proposed as an alternative to Mediterranean diets for their greater ability, at least in the short term, to induce body weight loss and improve glucose homeostasis by exploiting the beneficial actions of proteins [14,15] while reducing carbohydrate intake, which is among the main determinants of postprandial hyperglycemia. In a randomized clinical trial [8], we compared the short-term effects of a low-carbohydrate, high-protein diet with a Mediterranean diet matched for energy restriction in morbidly obese individuals at high risk of developing diabetes. The two dietary regimens proved similarly effective in improving insulin resistance, model-derived β-cell function, and insulin clearance. However, compared with the Mediterranean diet group, participants who were randomly assigned to the low-carbohydrate, high-protein diet achieved 60% greater body weight loss, reaching the target 5% weight reduction that has been proven effective in decreasing diabetes and cardiovascular risk. Adherence to the assigned diets in a free-living setting is a critical factor in the appraisal of studies comparing different nutritional interventions, as pointed out by Landry et al. [16] in their letter. Thus, we reported the significant endeavor undertaken to quantify and maximize participants’ engagement to both the proposed nutritional interventions in this clinical trial, ultimately aiming to reduce the influence of dietary adherence on study outcomes [17].

Boosting participants’ adherence and motivation to weight loss interventions, while increasing health benefits and economic sustainability, is extremely challenging. In their clinical trial, Driscoll and colleagues [10] integrated a telephone-delivered coaching service to multidisciplinary care for adults with obesity. Individual distance coaching provided high levels of patient acceptability and satisfaction, while delivering non-inferior changes in body weight and glycated hemoglobin. These findings indicate that telephone-delivered coaching may be adopted to improve patients’ access to obesity interventions without reducing the efficacy of such interventions.

The use of functional foods as an adjunct to lifestyle interventions represents a feasible and cost-effective strategy to improve the management of chronic diseases. Although an array of functional foods are available to offer nutritional support to individuals with diabetes, the glucose-lowering efficacy of these products is often modest. In their translational research project, Chauhan et al. [9] employed artificial intelligence to predict and generate a functional ingredient from pea protein through hydrolysis. Subsequently, the actions of this new ingredient, named NRT_N0G5IJ, were thoroughly tested in human skeletal muscle cells, db/db diabetic mice, and non-diabetic humans. Interestingly, treatment with NRT_N0G5IJ increased glucose uptake by skeletal muscle cells in vitro and reduced glycated hemoglobin in both animal and clinical studies, providing robust evidence of its glucose-lowering efficacy.

Along with food quantity and quality, the daily distribution of energy and carbohydrate intake has a key role in the regulation of body weight and glucose metabolism [6]. In their accurate review, Jakubowicz et al. [11] summarized the recent literature highlighting the positive effects of dietary patterns characterized by a high-energy breakfast, in which most carbohydrates are consumed in the early hours of the day, on weight loss and postprandial glycemic excursions. These actions, independent of total energy intake, are mediated by the alignment of carbohydrate intake to daily oscillations of the circadian clock, which has well-documented resetting and synchronizing actions on the expression of circadian clock genes.

Finally, Borg and colleagues [12] reviewed the pleiotropic actions of gut hormones, including glucagon-like peptide-1, glucose-dependent insulinotropic peptide, and somatostatin, in the modulation of the physiologic hemodynamic responses to ingested meals. Alterations in nutrient–gut interactions and associated neurohormonal responses can impair the compensatory cardiovascular responses to meal-induced splanchnic blood pooling, leading to recurrent postprandial hypotension. This overlooked pathological condition is associated with an increased risk for cardiovascular events and mortality [18], which may be mitigated by targeting the signaling pathways of gut peptides via nutritional or pharmacological interventions.

The studies featured in this Special Issue are critical to advancing our understanding of how dietary factors can provide support for the clinical management of chronic diseases, warranting exploration of the complex interplay between nutrient quantity, quality, and timing on cardiometabolic outcomes. Deciphering the mechanisms by which nutrition impacts glucose homeostasis and the cardiovascular system may reveal innovative approaches for personalized dietary interventions that promote optimal glycemic control and improved clinical outcomes in individuals with or at risk for type 2 diabetes.
